# Functional divergence of the brain-size regulating gene *MCPH1* during primate evolution and the origin of humans

**DOI:** 10.1186/1741-7007-11-62

**Published:** 2013-05-22

**Authors:** Lei Shi, Ming Li, Qiang Lin, Xuebin Qi, Bing Su

**Affiliations:** 1 State Key Laboratory of Genetic Resources and Evolution, Kunming Institute of Zoology, Chinese Academy of Sciences, 32 East Jiao-Chang Road, Kunming, Yunnan 650223, PR China; 2 University of the Chinese Academy of Sciences, No.19A Yuquan Road, Shijingshan District, Beijing, 100049, China

**Keywords:** *MCPH1*, *E2F1*, Brain size, Primates, Evolution, Functional divergence

## Abstract

**Background:**

One of the key genes that regulate human brain size, *MCPH1* has evolved under strong Darwinian positive selection during the evolution of primates. During this evolution, the divergence of *MCPH1* protein sequences among primates may have caused functional changes that contribute to brain enlargement.

**Results:**

To test this hypothesis, we used co-immunoprecipitation and reporter gene assays to examine the activating and repressing effects of *MCPH1* on a set of its down-stream genes and then compared the functional outcomes of a series of mutant *MCPH1* proteins that carry mutations at the human- and great-ape-specific sites. The results demonstrate that the regulatory effects of human *MCPH1* and rhesus macaque *MCPH1* are different in three of eight down-stream genes tested (*p73*, *cyclinE1* and *p14*^*ARF*^), suggesting a functional divergence of *MCPH1* between human and non-human primates. Further analyses of the mutant *MCPH1* proteins indicated that most of the human-specific mutations could change the regulatory effects on the down-stream genes. A similar result was also observed for one of the four great-ape-specific mutations.

**Conclusions:**

Collectively, we propose that during primate evolution in general and human evolution in particular, the divergence of *MCPH1* protein sequences under Darwinian positive selection led to functional modifications, providing a possible molecular mechanism of how *MCPH1* contributed to brain enlargement during primate evolution and human origin.

## Background

A dramatic increase in brain size is one of the hallmarks of human evolution, but despite the significance of this trait, the causal molecular mechanism underlying this expansion is unclear [1]. Until recently, addressing this question with genetic tools has been difficult because the dramatically enlarged brain is a human-specific trait. Genetic studies of a rare brain developmental disorder, human autosomal primary microcephaly syndrome (MCPH, OMIM251200), have uncovered a set of genes that regulate brain development. To date, seven genes have been identified as being responsible for this syndrome: *MCPH1*, also known as *BRIT1* (BRCT-repeat inhibitor of *hTERT* expression) [2,3], *WDR62* (WD repeat domain 62; *MCPH2*) [4-6], *CDK5RAP2* (cyclin-dependnet kinase 5 regulatory associated protein 2; MCPH3) [7], *CEP152* (centrosomal protein 152 kDa; MCPH4) [8], *ASPM* (abnormal spindle like microcephaly associated protein; MCPH5) [9], and *CENPJ* (centromeric protein J; MCPH6) [7] and *STIL* (SCL/TAL1 interrupting locus; MCPH7) [10].

Previous evolutionary analyses of these microcephaly genes showed that four of them, *ASPM, CDK5RAP2, CENPJ* and *MCPH1*, evolved rapidly under Darwinian positive selection during the evolution of human and non-human primates [11-16]. *ASPM* also experienced positive selection across anthropoids [14-16], while *CDK5RAP2* and *CENPJ* showed accelerated rates of non-synonymous substitutions over the course of primate evolution [11,16]. The signal of positive selection on *MCPH1* was observed in the common ancestor of great apes and humans as well as in the human lineage [12], although another study on *MCPH1* only detected positive selection in the anthropoids as a whole and no particular acceleration in the human lineage [16]. This rapid evolution suggests these genes may have had a key role in the evolutionary enlargement of the brain, although the link of *CENPJ* and *MCPH1* to the evolution of gross brain size was not confirmed in the association analysis of absolute neonatal brain size among primates [16]. Among the four rapidly evolving microcephaly genes, only *ASPM* has been experimentally studied to detect the evolutionary consequence of protein sequence changes; mice carrying a truncated ASPM protein were shown to have reductions of both brain and testis size, while the transgenic mouse carrying human ASPM could rescue this phenotype, but did not cause any additional enlargement of the brain [17].

*MCPH1* was the first gene identified as being responsible for autosomal recessive primary microcephaly, characterized by significantly reduced brain volume, mental retardation and premature chromosome condensation (PCC) syndrome [2,18]. The *MCPH1* gene encodes a 2,508-bp-long coding sequence (CDS) with 14 exons, spanning about 240 kb at 8p23. The *MCPH1* protein contains three BRCA1-Carboxyl Terminal (BRCT) domains, including one N terminal BRCT domain and a tandem pair at the C terminus. Numerous studies have implied that the BRCT domains of *MCPH1* function as the key component for protein-protein interaction; this seems likely as the interaction of the *MCPH1* tandem BRCT domains with proteins like *E2F1* and r-H2AX is required for the activation of cell cycle checkpoint, DNA repair and apoptosis [19-23]. Several studies have likewise suggested that *MCPH1* may also function as a tumor suppressor [3,23].

Evolutionary studies of *MCPH1* have demonstrated a rapid change in protein sequence associated with the brain enlargement during primate evolution and human origin. Interestingly, during two key taxonomic transitions in primates, that is, between lesser apes and great apes, and between great apes and humans, absolute brain volume was greatly enlarged, and *MCPH1* might be involved in this process [12]. Additionally, *MCPH1* is also highly polymorphic in human populations and still carries the molecular signature of on-going positive selection [12]. Human population studies have reported a sex-specific association between a *MCPH1* sequence variant and brain volume [24,25]. These results suggest that the protein sequence changes, especially the human-specific changes of *MCPH1* may have caused the functional changes that explain the genetic basis for the evolution of brain size in primates.

Previously, the *MCPH1* protein has been shown to play an essential role during cell cycle and cell apoptosis and it can physically interact with *E2F1* to form a complex and bind the promoters of the target genes for regulating their transcriptional activities [19]. Beyond this, *MCPH1* alone can also function as a transcriptional regulator, and we previously demonstrated *MCPH1* could function as a transcriptional repressor [26]. Together, these regulatory mechanisms allow the experimental testing of the functional changes of *MCPH1* during primate evolution.

To detect if the protein sequence divergence of *MCPH1* among primates may confer any functional alterations, we selected eight known down-stream genes regulated by *E2F1* and *MCPH1*: *p73*[19], *p107*[19], *p18*[27], *p27*[28], *p14*^*ARF*^[19], *Caspase7*[19], *CyclinE1*[19] and *hTERT*[26]. These genes are involved in cell proliferation and apoptosis, critical processes regulating brain development (see Additional file 1: Figure S1). We tested the activating effects (together with *E2F1*) and the repressing effects (*MCPH1* alone) of *MCPH1* on these genes’ promoter when introducing mutations at the sites containing human- and great-ape-specific amino acid changes. Our results demonstrated that most of the human-specific amino acid substitutions could influence the regulatory effects of *MCPH1* on the down-stream genes, and a similar effect was also seen for one of the four great-ape-specific changes, suggesting that the species and lineage specific mutations of *MCPH1* are indeed functional and potentially contributed to brain enlargement over the course of primate evolution.

## Results

### Identification of lineage-specific *MCPH1* amino acid substitutions

**Figure 1:**
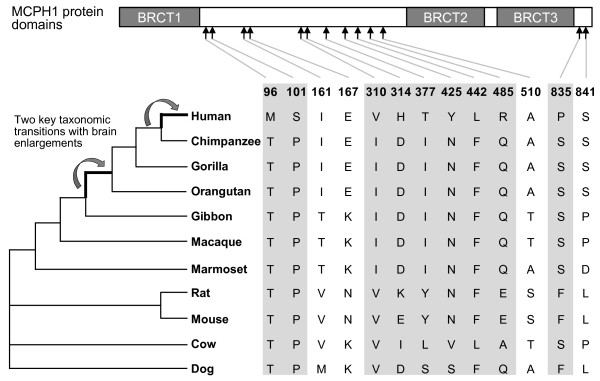
**Schematic map of *MCPH1* protein domains labeled with lineage-specific amino acid substitutions.** Sites containing human specific substitutions are marked with shadows, and the two dramatic brain size enlargement events that coincided with the molecular signatures of Darwinian positive selection during primate evolution are indicated.

In order to identify lineage-specific amino acid substitutions, *MCPH1* orthologs of representative primate and other mammalian species were obtained from the NCBI, EMBL and UniProt databases. We used a total of 11 species including 7 primates (human, great apes, lesser ape, Old World monkey and New World monkey) and 4 other representative mammalian species (mouse, rat, dog and cow) (Figure 1). Using MUSCLE and Clustal W, we aligned the *MCPH1* protein sequences (see Additional file 2: Figure S2). During primate evolution, there are two key taxonomic transitions accompanied by brain enlargements (between lesser apes and great apes, and between great apes and humans). We identified nine sites containing substitutions considered as human-specific sites that occur in the human lineage but are relatively conserved in the other species (M96, S101, V310, H314, T377, Y425, L442, R485 and P835; Figure 1, Additional file 3: Table S1). We further selected four sites containing substitutions considered as great-ape-specific since they occurred in the ancestor of Hominidae (I161, E167, A510 and S841; Figure 1, Additional file 3: Table S1). The physicochemical properties of the 13 lineage-specific amino acid substitutions are shown in Additional file 3: Table S1 and the schematic map of the *MCPH1* protein domains labeled with the lineage-specific substitutions are shown in Figure 1. All these lineage-specific sites were selected to generate mutant *MCPH1* proteins for the reporter gene assays in order to test their functional effects.

### Test of protein-protein interaction between *MCPH1* and *E2F1*

**Figure 2:**
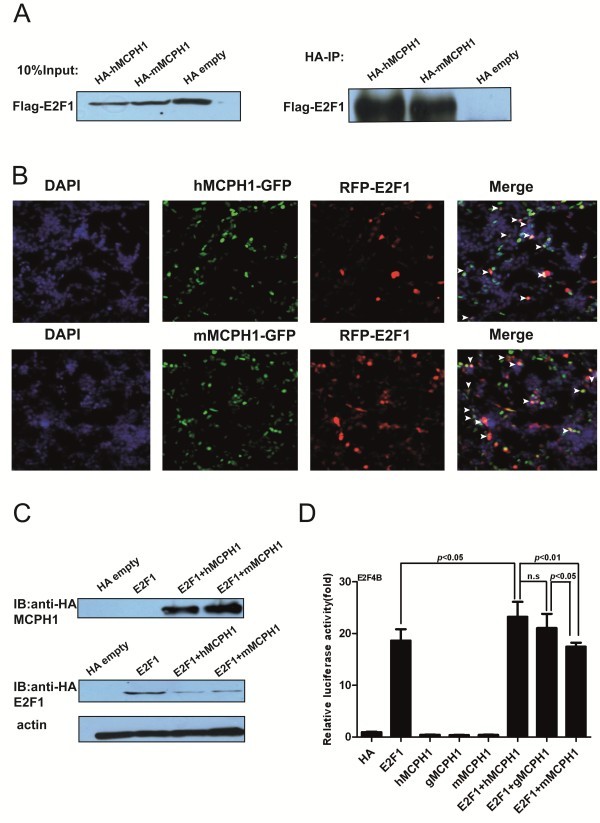
**Human and macaque *MCPH1* can directly interact with *E2F1*.** (**A**) Interaction of the transfected *E2F1* and *MCPH1* in HEK293T cells. (**B**) Co-localization of *MCPH1*-GFP and RFP-*E2F1* in HEK293T cells. White arrows indicate co-staining areas. (**C**) Expression of the HA-tagged human and macaque *MCPH1*s were verified by Western blot using anti-HA monoclonal antibody (top panel), and the HA-tagged *E2F1* was also verified by Western blot (middle panel). The bottom panel is the actin control. (**D**) Quantification of *E2F1* and *MCPH1* transcriptional activity using the E2F4B constructs. All histograms represent the mean ± SD of at least nine data points, including both biological and technical replicates (* *P* <0.05,** *P* <0.01, *** *P* <0.001, n.s, not significant).

Previous studies suggested human *MCPH1* (*hMCPH1*) could interact with *E2F1in vitro* and *in vivo*[19]. To test if the non-human primate *MCPH1* can also interact with *E2F1*, we cloned the rhesus macaque *MCPH1* (*mMCPH1*). The results of the co-immunoprecipitation assay showed that mMPCH1 can also directly interact with *E2F1*, and no difference was observed for the intensity of the interaction with *E2F1* between *hMCPH1* and *mMCPH1* (Figure 2A). Given the established *MCPH1*-*E2F1* interaction in human cell lines [19], this protein-protein interaction mechanism seems likely to have been conserved during primate evolution. We also conducted a cellular co-localization assay for both *mMCPH1* and *hMCPH1*, and the results indicated that both were co-localized with *E2F1* (Figure 2B), consistent with the results of the co-immunoprecipitation assay.

### Divergent effects of *hMCPH1* and *mMCPH1* on transcriptional regulation

*MCPH1* interacts with *E2F1* to enhance its transactivation activity by forming a complex, which binds to the promoters of the *E2F1* target genes, including *p73*, *Caspase* and *RAD51*, among others [19]. We, therefore, tested the enhancing effect of *MCPH1* using the E2F4B reporter vector commonly used as the positive control in testing the transactivation activity of *E2F1*[29]. The expression of the HA-tagged *MCPH1* and *E2F1* were verified by Western blot using anti-HA monoclonal antibodies (Figure 2C). Our results showed that *hMCPH1* could significantly enhance the transactivation activity of *E2F1*, but no significant effect was observed for *mMCPH1* and *gMCPH1* (the gibbon copy of *MCPH1*) (Figure 2D). In addition, the difference between *E2F1* + *hMCPH1* and *E2F1* + *mMCPH1* is significant (*P* <0.01, ANOVA test). The same trend was also seen when comparing *E2F1* + *hMCPH1* and *E2F1* + *gMCPH1*, though not statistically significant (Figure 2D). Interestingly, the difference between *E2F1* + *gMCPH1* and *E2F1* + *mMCPH1* is significant (*P* <0.05, ANOVA test), suggesting continuum of functional divergence of *MCPH1* during primate evolution with a major shift in function between Old World monkeys and apes and a probable further shift during human evolution (Figure 2D).

To check the conservation of the *E2F1* binding sites (CGCGC), we aligned the promoter sequences of the target genes among representative primate species. We found that most of the *E2F1* binding sites are highly conserved from humans to marmosets (see Additional file 4: Figure S3), ruling out the possibility of binding bias due to promoter sequence divergence. The *E2F1* protein sequences are also highly conserved among primate species (Additional file 5: Figure S4), ruling out the possibility that the different effect of *MCPH1* is caused by mutations in *E2F1*. Collectively, these results suggest a functional divergence of *MCPH1* between humans and nonhuman primates.

**Figure 3:**
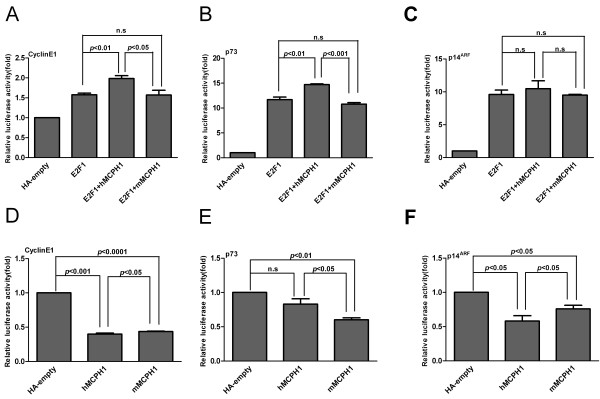
**Comparison of *MCPH1* enhancing and repressing effects between humans and macaques.** (**A**) The enhancing effect difference between *hMCPH1* and *mMCPH1* on the *CyclinE1* promoter. (**B**) The enhancing effect difference between *hMCPH1* and *mMCPH1* on the *p73* promoter. (**C**) The enhancing effect difference between *hMCPH1* and *mMCPH1* on the *p14*^*ARF*^ promoter. For enhancing effect assay, 0.2 ug target gene promoter construct was co-transfected with 0.2 ug HA tagged *E2F1* per/well or 0.2 ug HA tagged *E2F1* and 0.2 ug HA tagged *hMCPH1* per/well or 0.2 ug HA tagged *E2F1* and 0.2 ug HA tagged *mMCPH1* per well in 24-well plates in HEK923T cells, Renilla was used as the internal control. The HA-tagged empty vector was used as the negative control. (**D**) The repressing effect difference between *hMCPH1* and *mMCPH1* on the *CyclinE1* promoter. (**E**) The repressing effect difference between *hMCPH1* and *mMCPH1* on the *p73* promoter. (**F**) The repressing effect difference between *hMCPH1* and *mMCPH1* on the *p14*^*ARF*^ promoter. For repression assay, 0.2 ug target gene promoter construct was co-transfected with 0.2 ug HA tagged *hMCPH1* per/well or 0.2 ug HA tagged *mMCPH1* per/well in 24-well plates in HEK923T cells, Renilla was used as the internal control. The HA-tagged empty vector was used as the negative control. All histograms represent the mean ± SD of at least nine data points including both biological and technical replicates (* *P* <0.05,** *P* <0.01, *** *P* <0.001, n.s, not significant).

We further tested a set of *E2F1* target genes (*p73*, *p107*, *p18*, *p27*, *p14*^*ARF*^, *Caspase7* and *CyclinE1*). The regulatory network of these genes is shown in Additional file 1: Figure S1. We found that the enhancing effect of *hMCPH1* was significantly stronger than *mMCPH1* for *CyclinE1* and *p73* (Figure 3A, B) and a similar trend was also observed for *p14*^*ARF*^, though not significantly (Figure 3C). No significant enhancing effects were detected for the other four genes, *p18*, *p27*, *p107* and *Caspase7* (see Additional file 6: Figure S5). Consistent with the result from the E2F4B assay, these data also suggest a functional divergence between *hMCPH1* and *mMCPH1* in enhancing the transactivation activity of *E2F1*.

To further test the functional divergence between *hMCPH1* and *mMCPH1*, we performed another assay to detect the regulatory effect of *MCPH1* alone on eight down-stream genes (*p73*, *p107*, *p18*, *p27*, *p14*^*ARF*^, *Caspase7*, *CyclinE1* and *hTERT*). Among the eight genes tested, seven showed a repressing effect for both *hMCPH1* and *mMCPH1*, while only one, *Caspase7*, showed an activating effect. When comparing the effects between *hMCPH1* and *mMCPH1*, three genes (*CyclinE1*, *p73* and *p14*^*ARF*^) showed significant differences (*P* <0.05) of repressing effect (Figure 3D--F), the same three which showed enhancing differences between *hMCPH1* and *mMCPH1* in the *E2F1*-*MCPH1* transactivation assay (Figure 3A-C). For *CyclinE1* and *p14*^*ARF*^, *hMCPH1* showed a stronger repressing effect than *mMCPH1*, while the opposite effect was observed for *p73*. We did not detect significant between-species differences for the other four genes with repressing effect (*p18*, *p27*, *p107* and *hTERT*) though the trend was the same (see Additional file 7: Figure S6). *Caspase 7* was the only gene with an activating effect, and we also observed a significant difference between *hMCPH1* and *mMCPH1* (*P* <0.05) (see Additional file 7: Figure S6D). Collectively, the data from the two reporter gene assays suggest a clear functional divergence of *MCPH1* between humans and rhesus macaques.

### Detection of regulatory changes of *MCPH1* for the human-specific sites

**Figure 4:**
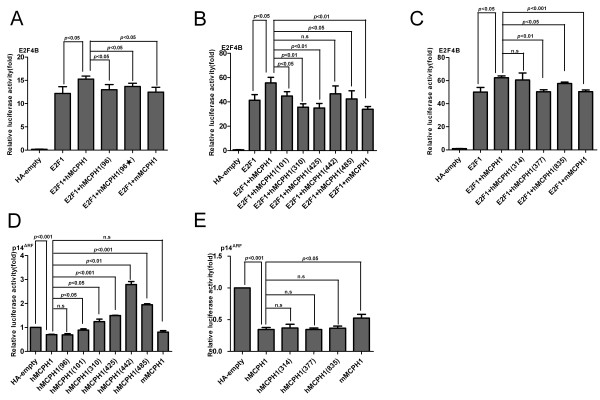
**Results of assays testing transcriptional regulations of mutant *MCPH1*s containing mutations at human-specific sites.** (**A-C**) The results of the E2F4B assay testing the changes of enhancing effect. (**D-E**) The results of the *p14*^*ARF*^ assay testing the repressing effect. All histograms represent the mean ± SD of at least nine data points including both biological and technical replicates (* *P* <0.05,** *P* <0.01, *** *P* <0.001, n.s, not significant).

In order to test the functional effect of the human specific amino acids changes, we first constructed two mutant *hMCPH1*s by introducing a backward mutation of changing the human-specific amino acid to an ancestral amino acid (Met96Thr) and introducing a random mutation (Met96Leu) at Site-96 (Figure 1). With the use of the E2F4B transactivation reporter assay, we measured the enhancing effect of the two mutant *hMCPH1*s. As shown in Figure 4A, both mutant *hMCPH1*s showed a significantly decreased enhancing effect as compared with the wild-type *hMCPH1*, similar to the difference between *hMCPH1* and *mMCPH1*, suggesting that the human-specific amino acid change at Site-96 can influence the enhancing effect of *MCPH1*.

We next generated a series of mutant *hMCPH1*s carrying backward mutations at the other eight human-specific sites (101, 310, 314, 377, 425, 442, 485 and 835), and tested their enhancing effects. Five of the eight mutant *hMCPH1*s (101, 310, 377, 425 and 835) showed significantly decreased enhancing effects compared with the wild-type *hMCPH1* (*P* <0.05) (Figure 4B, C) and the three non-significant sites (314, 442 and 485) showed the same trend. Among the nine human specific sites tested, the majority (6/9) showed a significantly decreased enhancing effect when mutated to the ancestral amino acids, suggesting most of the human-specific *MCPH1* amino acid changes are in fact functional.

We also tested the repressing effect on *p14*^*ARF*^ for the human-specific sites. As shown in Figure 4D, E, we detected a significant decrease of the *p14*^*ARF*^ repressing effect for five sites (101, 310, 425, 442 and 485), and the same trend was observed for the other four, though it was not significant. Among the five significant sites, three (101, 310 and 425) overlapped with the six sites showing decreased enhancing effect. Taken together, we detected regulatory changes for most (eight out of nine) human-specific sites, suggesting *hMCPH1* has acquired functional modifications during the origin of humans.

### Detection of regulatory changes of *MCPH1* for the great-ape-specific sites

**Figure 5:**
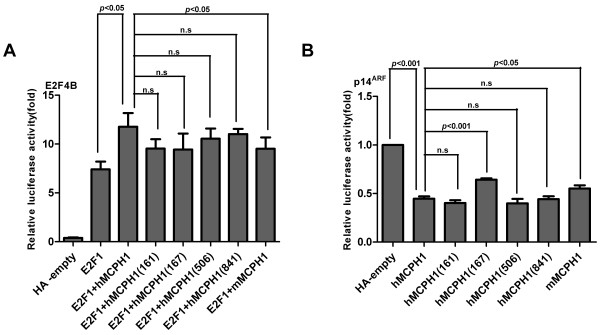
**Results of assays testing transcriptional regulations of mutant *MCPH1*s containing mutations at human-specific sites.** (**A**) Results of the E2F4B assay testing the changes of enhancing effect. (**B**) Results of the *p14*^*ARF*^ assay testing the repressing effect. All histograms represent the mean ± SD of at least nine data points, including both biological and technical replicates (* *P* <0.05,** *P* <0.01, *** *P* <0.001, n.s, not significant).

To detect the functional consequences of *MCPH1*’s four great-ape-specific mutations (Figure 1), we conducted analyses similar to that used for the human-specific sites. The results indicated that for the enhancing assay, although all four great-ape-specific sites showed a decreased level as compared with the wild-type of *hMCPH1*, none were statistically significant (*P* >0.05; Figure 5A). In contrast, in the repressing assay, one great-ape-specific site (Site-167) showed a significant decrease of the repressing effect (Figure 5B), suggesting that this great-ape-specific mutation may have also caused functional modifications of *MCPH1* during the evolution of the ancestor of Hominidae.

## Discussion

*MCPH1* has experienced strong Darwinian positive selection during primate evolution, but there are no data showing functional divergence. Here we demonstrated evidence of functional divergence of *MCPH1* between humans and nonhuman primates. Most of the human-specific amino acid changes could alter the regulatory effects of *MCPH1* on the transcription of the down-stream genes, and a similar effect was observed for one of the four great-ape-specific amino acid changes. Accordingly, our data support the hypothesis that selection on *MCPH1* has resulted in functional divergence at the protein level, which potentially contributes to changes in the development and evolution of brain size.

Absolute brain size has increased in parallel across primate evolution. Along the two branches we focused on in this analysis, absolute brain volume increased from 70 to 152 ml to 230 to 565 ml during the transition between lesser apes and great apes [30,31], and from 230 to 565 ml to 1,129 to 1,685 ml during the transition between great apes and humans [30,31]. Previous studies indicated that there were accelerated amino acid substitutions during both the origin of Hominidae’s ancestor and of our own species, paralleling the two brain enlargements [12], suggesting that the amino acid substitutions of *MCPH1* were probably adaptive and may have contributed to the brain expansion during primate evolution. In addition, the gradient change of *MCPH1*’s transcription regulation from macaque to gibbon, and to humans (Figure 2D) seems to imply a continuum of functional divergence rather than a number of discrete shifts, which calls for further functional tests in extensive primate lineages.

Interestingly, all the human- and great-ape-specific mutations are located in the non-BRCT domains (Figure 1). Since the three BRCT domains of *MCPH1* are critical for protein-protein interaction, the amino acid changes during primate evolution seems not to have caused drastic functional alteration, but rather a modification of the existing function.

Inferring the exact functional alterations of the human-specific and great-ape-specific mutations is difficult. Previous studies have shown that the middle domain (residues 367 to 485) of *MCPH1*, where the four human-specific mutations are located, is the binding domain by *Condensin II* for homologous recombination repair [32,33], an important mechanism for cell cycle checkpoints and genome integrity. Concurrently, all four human-specific sites located in this middle domain showed altered regulatory effects when mutated into ancestral amino acids, suggesting that the human-specific mutations may have changed the binding property with *Condensin II*. Additionally, all four human-specific mutations caused changes in physicochemical properties of amino acids (see Additional file 3: Table S1).

We also found that for the regulatory changes of the down-stream genes, almost half (three out of eight) of the tested genes (*p73*, *CyclinE1* and *p14*^*ARF*^) had significant differences between humans and rhesus macaques, either in the enhancing assay with *MCPH1*-*E2F1* or in the repressing assay with *MCPH1* alone (Figure 3), indicating a functional divergence between humans and non-human primates. The protein *p73* is involved in both cell cycle regulation and induction of apoptosis [34]. *E2F1* is an important regulator of *p73*, especially during brain development [35]. *CyclinE1*, meanwhile, is involved in cell cycle and is a key target gene of *E2F1*[36] and has been shown to take part in the determination of the number of neurons during mouse corticogenesis by regulating the G1 mode of cell division [37]. *p14*^*ARF*^ is an alternate reading frame product of CDKN2A involved in cell cycle regulation that is also involved in self-renewal of neural stem cells and neural development [38-41]. Additionally, human population studies have reported that the *MCPH1* sequence variants were associated with brain volume in a sex-specific manner [24,25]. Recently, it was also reported that *MCPH1* might have contributed to the evolution of sexual dimorphism in brain mass across anthropoid primates [42]. In fact, two of the down-stream genes regulated by *MCPH1*, *p73* and *cyclinE1*, were reported to be associated with sex dimorphism during germ line development [43,44], suggesting that the regulation of *MCPH1* on brain development may differ between males and females. Taken together, the strengthened transactivation effect of human *MCPH1* on these down-stream genes may contribute to the greatly enlarged neuro-progenitor pool in the human brain during neurogenesis, which is in line with recent studies that suggest *MCPH1*’s functional role in neuro-progenitor cells through the Chk1-Cdc25-Cdk1 pathway [45,46].

Conversely, when *MCPH1* acts alone as a transcription repressor, there were also differences between humans and rhesus macaques on the repressing effect of the down-stream genes (*p73*, *CyclinE1* and *p14*^*ARF*^), implying that a homeostasis of gene expression regulation by *MCPH1* is required during neurogenesis.

Although we observed functional divergence of *MCPH1* due to its protein sequence changes during primate evolution and human origin, it should be stressed that we did not establish a direct link between the adaptive changes of *MCPH1* and the ever-increasing brain size in primates. As shown in the *MCPH1* knock-out mice analysis, the truncated *MCPH1* not only caused a reduction in brain size, but also resulted in a reduction of testis size [45], suggesting that *MCPH1* may also play a role during testis development. Accordingly, we cannot rule out the possibility that the adaptive evolution of *MCPH1* in primates may be caused by selection on other phenotypes, though the current evidences mostly favor enlargement of the brain.

Initially proposed by King and Wilson [47], the importance of cis-regulatory changes in human evolution has recently been tested and confirmed [48]. However, our functional data of *MCPH1* suggests that protein sequence changes may also have significant phenotypic effects. Hence, the evolution of an important trait like brain function may require genetic alterations at multiple regulatory levels.

## Conclusions

We demonstrated the existence of functional alterations caused by the lineage-specific mutations of *MCPH1* during the evolution of primates, especially during the origin of humans. The functional changes of *MCPH1* are likely executed by regulating several key down-stream genes.

## Methods

### Ethical statement

The research protocol of this study was approved by the internal review board of Kunming Institute of Zoology, Chinese Academy of Sciences (Approval ID: SYDW-2012011).

### Cell culture

The HEK293T cell line was obtained from ATCC. Cells were cultured in Dulbecco’s Modified Eagle Medium (DMEM) (Gibco, Rockville, MD, USA) with 10% fetal bovine serum (Hyclone, Logan, UT, USA) at 37°C in a humidified atmosphere containing 5% CO_2_.

### Cloning of the macaque and gibbon *MCPH1* gene

To clone the cDNA of the macaque and gibbon *MCPH1* gene, we extracted the total RNA from macaque and gibbon brain tissue using TRIzol (Invitrogen, Carlsbad, CA, USA). RACE was carried out using a SMART^TM^ RACE cDNA amplification kit (Clontech, Palo Alto, CA, USA). The nested PCR was used and the primer sequences are:

macaMCPH1-3RACE: 5GAGAAAGAGGAGCATCAGGAGATCTATCA3;

macaMCPH1-5RACE: 5GGATTCCTCAGAAGTCACGCAACTGA3;

macaMCPH1-nest_3RACE: 5GAAAGAGGAGCATCAGGAGATCTATCAT3;

macaMCPH1-nest_5RACE: 5CAGAAGTCACGCAACTGAAAGTTGCA3

gibbonMCPH1-3RACE: 5TTAGCTGTGGGGAGTCTTCATATGATGAC3;

gibbonMCPH1-5RACE: 5GCGGGGTCCTCAATGGTGTAAGA3;

gibbonMCPH1-nest_3RACE: 5AGCTGTGGGGAGTCTTCATATGATGAC3;

gibbonMCPH1-nest_5RACE: 5GGTCCTCAATGGTGTAAGAAAAGCCA3;

The macaque *MCPH1* PCR products were cloned into the pMD-19 simple-T-vector (Takara, Tokyo, Japan), then digested with BamH I and EcoR I, and cloned into a pCGN-HAM vector. The gibbon *MCPH1* PCR products were cloned into the pMD-19 simple-T-vector (Takara, Tokyo, Japan), then digested with HindIII and AgeI, and cloned into a pCGN-HAM vector. The final constructs were confirmed by sequencing (ABI-3130 automatic sequencer).

### Cloning of fluorescent *MCPH1* plasmids

The full length cDNAs of human *MCPH1* and macaque *MCPH1* were PCR amplified, and the PCR products were digested with Age I and EcoR I and cloned into frame for N-terminal fusions into the cFUGW plasmids. The final constructs were confirmed by sequencing (ABI-3130 automatic sequencer).

### Transient transfection and luciferase reporter assays

All transfections were carried out in triplicates in the 24-well plates (Corning, Corning, NY, USA). About 2 × 10^5^ cells were seeded for 24 h prior to transfection. Briefly, equal numbers of cells were plated in the 24-well and 6-well plates and grown to 80% confluence. The indicated amounts of vectors were mixed in OPTI-MEM medium (Gibco) with Lipofectamine 2000 (Invitrogen). The solution was incubated for about 30 minutes at room temperature and then placed on the cultured cells. After four to six hours, the medium was changed into DMEM (Gibco) with 10% fetal bovine serum (Hyclone). For luciferase assay, cells were grown in the 24-well plates and transfected with the indicated amounts of vectors, including pTK-*Renilla* as an internal control, and Lipofectamine 2000 (Invitrogen) was used. Luciferase activity was assayed 28 to 32 h after transfection. The luciferase activity in cell extracts was determined by the Dual-luciferase Reporter Assay System (Promega, Madison, WI, USA) according to the manufacturer’s protocol. The relative light units were measured using a luminometer.

The wide range of relative luciferase activity seen in different panels was likely due to the different amount of cells used at each biological replicate, which did not influence the measurement of relative activity. At least three technical replicates were conducted for each experiment. To avoid transfection efficiency bias, we also performed at least three biological replicates for the luciferase assay. The promoter constructs of *p73*, p107, p18, p27, *p14*^*ARF*^, Caspase7 and *CyclinE1* were kindly provided by Dr. Wuhan Xiao from Institute of Hydrobiology, Chinese Academy of Sciences, and these constructs were published before [26,49,50].

To test the co-localization of *MCPH1* and *E2F1*, the GFP tagged *MCPH1* expression vector was co-transfected with the RFP tagged *E2F1* expression vector into the HEK293T cells. After 24 to 48 h, the cells were checked under fluorescent microscopy.

### Generating *MCPH1* mutants

The human *MCPH1* gene copy was used to generate mutants carrying mutations at the sites with human-specific and great-ape-specific mutations. A total of 13 sites were tested, including the 9 sites containing human-specific mutations and the 4 sites containing great-ape-specific mutations. The mutant *MCPH1*s were prepared using the QuickChange II XL site-directed mutagenesis kit (Stratagene, La Jolla, CA, USA) and specific oligodeoxynucleotide primer sets. The intended mutations were confirmed by sequencing. The oligodeoxynucleotide primers used for generating the mutant *MCPH1*s are shown in Additional file 8: Table S2 and Additional file 9: Table S3.

### Co-immunoprecipitation

Flag-*E2F1* and HA-*MCPH1* were co-transfected into the HEK293T cells in the six-well plates by Lipofectamine 2000 with the total amount of 16 ug DNA. After 36 to 38 h of transfection, cells were lysed with 400 ul lysis buffer (50 ml Tris–HCl, pH = 7.4; 150 mM NaCl; 1 mM EDTA; 1% Triton-100; 1 Mm Na_3_VO_4_) containing a cocktail of protease inhibitors (Sigma Chemical St. Louis, MO, USA). Cell lyses were incubated with HA agarose beads (Sigma Chemical) overnight at 4°C with lysis buffer, and boiled in 2 × protein loading buffer. Blots were blocked in 0.05% Tris-buffered saline (TBS), 20% Tween and 5% non-fat milk followed by incubation with the indicated primary (anti-Flag (M2) from Sigma or anti-HA from Covance, Princeton, NJ, USA) and secondary antibody (anti-mouse from KPL, Inc. Maryland, Washington, D.C, USA) in this buffer. Immunoreactivity was detected with an enhanced chemiluminescence system (Pierce Protein Biology, Rockford, IL) with colored markers (Fermentas, Pittsburgh PA, USA) as the molecular size standard.

### Western blotting

Proteins from the HEK293T cells were homogenized in RIPA lysis buffer (50 mM Tris–HCl, pH 7.4; 150 mM NaCl; 1 mM EDTA; 1% Triton-100; 1 mM Na_3_VO_4_) containing a cocktail of protease inhibitor (Sigma Chemical). Extracted proteins (15 to 20 μg) were separated by SDS-polyacrylamide gel electrophoresis and electrophoreticly transferred to a membrane incubated with anti-HA monoclonal antibody (Covance). Immunoreactivity was detected with an enhanced chemiluminescence system (Pierce Protein Biology) with colored markers (Fermentas) as the molecular size standard.

### *MCPH1* protein sequence comparison among representative mammalian species

The *MCPH1* protein sequences of human, non-human primates (chimpanzee, gorilla, orangutan, gibbon, macaque and marmoset) and representative vertebrate species (rat, mouse, cow and dog) were obtained from the NCBI [51], EMBL [52] and UniProt [53] databases. Orthologous sequences were aligned using Muscle in MEGA5 [54] and Clustal W 7.0.5.2 (BioEdit software. http://www.mbio.ncsu.edu/bioedit/bioedit.html. Tom Hall Ibis Bioscience Carlsbad, CA, USA) (see Additional file 2: Figure S2).

### Statistical analysis

Statistical analysis was performed using Prism 5 (GraphPad Software, Inc. 2236 Avenida de la Playa La Jolla, CA 92037 USA), the data were analyzed using the two-tailed Student’s *t* test. ANOVA test analysis using R program [55]. A *P*-value of <0.05 was considered statistically significant.

### Database

Nucleotide sequences have been deposited to the NCBI GeneBankTM database [56] with accession numbers JX194162 and JX861895 for the *MCPH1* coding region sequences of rhesus macaque and gibbon.

## Abbreviations

ASPM: Abnormal spindle like microcephaly associated protein; BRCT: BRCA1-carboxyl terminal; CDK5RAP2: Cyclin-dependnet kinase 5 regulatory associated protein 2; CDS: Coding sequence; CENPJ: Centromeric protein J; CEP152: Centrosomal protein 152 kDa; DMEM: Dulbecco modified eagle medium; IP: Immunoprecipitation; MCPH: Primary microcephaly; PCC: Premature chromosome condensation; STIL: SCL/TAL1 interrupting locus; TBS: Tris-buffered saline; TSS: Transcriptional start site; WDR62: WD repeat domain 62.

## Competing interests

The authors declare that no competing interests exist.

## Authors’ contributions

LS and BS designed the study. LS performed experiments. ML, QL and XBQ contributed analytic tools. LS and BS analyzed data. LS and BS wrote the paper. All authors read and approved the final manuscript.

## Supplementary Material

Additional file 1: Figure S1 Summary of the *E2F1* regulatory pathway. *E2F1* could up-regulate cell apoptosis associated genes *p73*, *p14*^*ARF*^, Caspase7 and cell proliferation associated genes *CyclinE1*, p107, p18 and p27 promoters’ activity. *E2F1* also represses the *hTERT* promoter activity. Click here for file

Additional file 2: Figure S2 Alignment of the full length *MCPH1* protein sequences among different species including human, chimpanzee, gorilla, orangutan, gibbon, macaque, marmoset, rat, mouse, cow and dog. The framed sites are the human- and great-ape-specific sites. Click here for file

Additional file 3: Table S1 The physicochemical properties of *MCPH1* lineage specific amino acids. Click here for file

Additional file 4: Figure S3 Alignment of the promoter sequences of *p73*, p107, p18, p27, *p14*^*ARF*^, Caspase7 and *hTERT* (genes’ transcriptional start site (TSS) downstream 500bp and TSS upstream 2000bp) among primate species including human, chimpanzee, gorilla, orangutan, macaque and marmoset. The aligned sequences are the *E2F1*-specific binding sites located in the promoter region. The promoter sequences of *CyclinE1* were not available for most of the nonhuman primate species. For Caspase 7, the promoter sequences of chimpanzee and marmoset were not available. The numbers for positions are the distances from the transcriptional start site. Click here for file

Additional file 5: Figure S4 Alignment of the full length *E2F1* protein sequences among different primate species including human, chimpanzee, gorilla and macaque. Click here for file

Additional file 6: Figure S5 The results of the enhancing assay for the *E2F1* target genes *p18*, *p27*, *p107* and *Caspase7*. Click here for file

Additional file 7: Figure S6 The results of the repressing assay for the target genes including *p18*, *p27*, *p107*, *Caspase7* and *TERT*. Click here for file

Additional file 8: Table S2 Primers used for the generation of human-specific mutants. Click here for file

Additional file 9: Table S3 Primers used for the generation of great-ape-specific mutants. Click here for file
