# Supplementary material for: Functional divergence of the brain-size regulating gene MCPH1 during primate evolution and the origin of humans
Source: BMC Biol. 2013 May 22;11:62. doi: 10.1186/1741-7007-11-62 (PMC3674976; doi:10.1186/1741-7007-11-62)
Supplement: Additional file 1: Figure S1 — Summary of the E2F1 regulatory pathway. E2F1 could up-regulate cell apoptosis associated genes p73, p14ARF, Caspase7 and cell proliferation associated genes CyclinE1, p107, p18 and p27 promoters’ activity. E2F1 also represses the hTERT promoter activity. [file 1741-7007-11-62-S1.docx]

**Figure S1.** Summary of the E2F1 regulatory pathway. E2F1 could up-regulate cell apoptosis associated genes p73, p14^ARF^, Caspase7 and cell proliferation associated genes CyclinE1, p107, p18 and p27 promoters’ activity. E2F1 also represses the hTERT promoter activity.

**
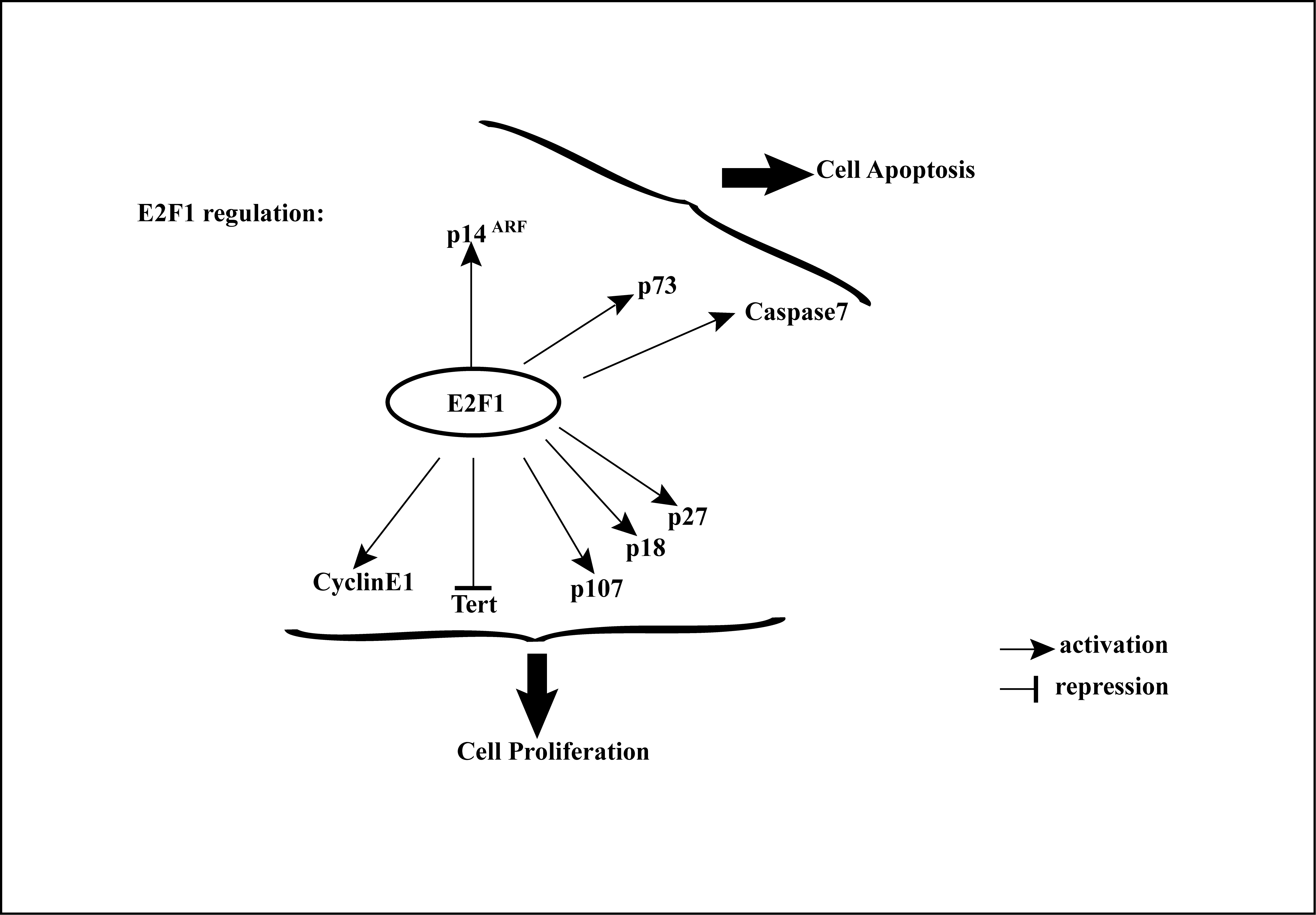
**
