# Supplementary material for: Functional divergence of the brain-size regulating gene MCPH1 during primate evolution and the origin of humans
Source: BMC Biol. 2013 May 22;11:62. doi: 10.1186/1741-7007-11-62 (PMC3674976; doi:10.1186/1741-7007-11-62)
Supplement: Additional file 2: Figure S2 — Alignment of the full length MCPH1 protein sequences among different species including human, chimpanzee, gorilla, orangutan, gibbon, macaque, marmoset, rat, mouse, cow and dog. The framed sites are the human- and great-ape-specific sites. [file 1741-7007-11-62-S2.docx]

**Figure S2.** Alignment of the full length MCPH1 protein sequences among different species including human, chimpanzee, gorilla, orangutan, gibbon, macaque, marmoset, rat, mouse, cow and dog. The framed sites are the human- and great-ape-specific sites.

**
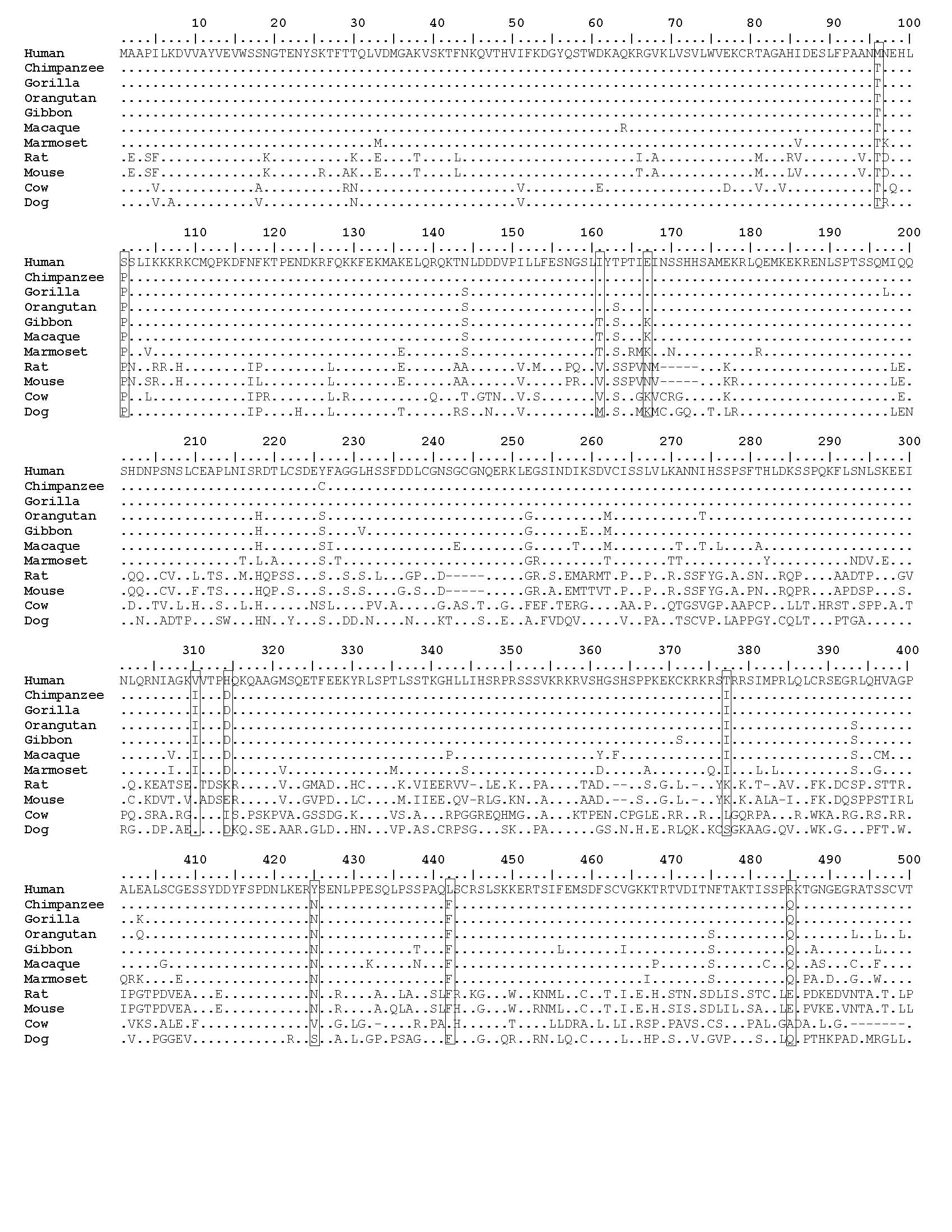
**

**
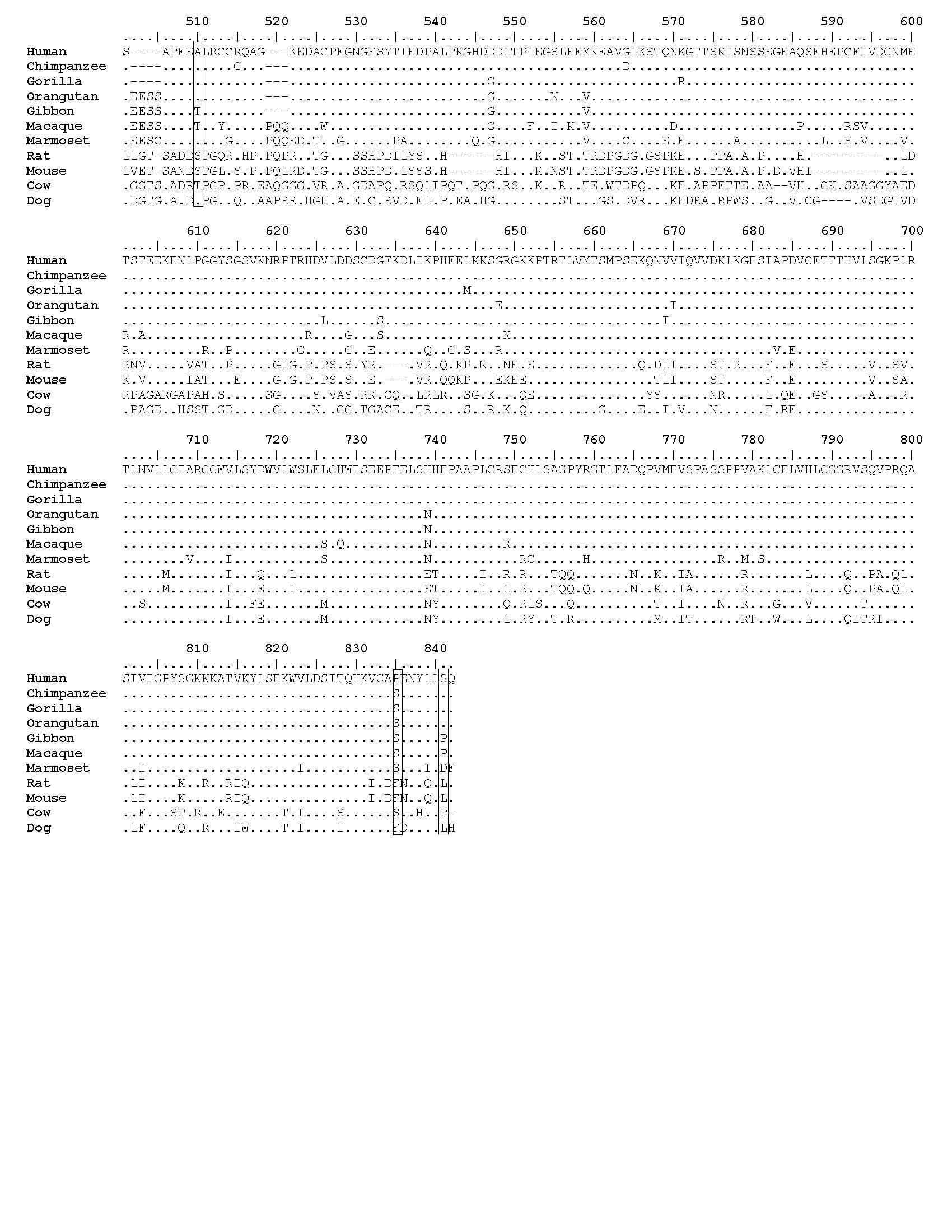
**
