# Supplementary material for: Functional divergence of the brain-size regulating gene MCPH1 during primate evolution and the origin of humans
Source: BMC Biol. 2013 May 22;11:62. doi: 10.1186/1741-7007-11-62 (PMC3674976; doi:10.1186/1741-7007-11-62)
Supplement: Additional file 3: Table S1 — The physicochemical properties of MCPH1 lineage specific amino acids. [file 1741-7007-11-62-S3.docx]

**Table S1.** The physicochemical properties of MCPH1 lineage specific amino acids.

| Position | Charge | |  | Polarity | |  | Volume | |
| --- | --- | --- | --- | --- | --- | --- | --- | --- |
|  | Lineage specific | Ancestral |  | Lineage specific | Ancestral |  | Lineage specific | Ancestral |
| 96 | Neutral | Neutral |  | Nonpolar | Polar |  | Relatively small | Small |
| 101 | Neutral | Neutral |  | Polar | Nonpolar |  | Small | Small |
| 310 | Neutral | Neutral |  | Nonpolar | Nonpolar |  | Relatively small | Relatively small |
| 161 | Neutral | Neutral |  | Nonpolar | Polar |  | Relatvively small | Small |
| 167 | Negative | Positive |  | Polar | Polar |  | Relatvively small | Relatively large |
| 314 | Positive | Negative |  | Polar | Polar |  | Relatively large | Relatively small |
| 377 | Neutral | Neutral |  | Polar | Nonpolar |  | Small | Relatively small |
| 425 | Neutral | Neutral |  | Polar | Polar |  | Relatively large | Relatively small |
| 442 | Neutral | Neutral |  | Nonpolar | Nonpolar |  | Relatively small | Relatively large |
| 485 | Positive | Neutral |  | Polar | Polar |  | Relatively large | Relatively small |
| 510 | Neutral | Neutral |  | Nonpolar | Polar |  | Small | Small |
| 835 | Neutral | Neutral |  | Nonpolar | Polar |  | Small | Small |
| 841 | Neutral | Neutral |  | Polar | NonPolar |  | Small | Small |

* The human specific substitutions are marked with shadows.
