# Supplementary material for: Functional divergence of the brain-size regulating gene MCPH1 during primate evolution and the origin of humans
Source: BMC Biol. 2013 May 22;11:62. doi: 10.1186/1741-7007-11-62 (PMC3674976; doi:10.1186/1741-7007-11-62)
Supplement: Additional file 4: Figure S3 — Alignment of the promoter sequences of p73, p107, p18, p27, p14ARF, Caspase7 and hTERT (genes’ transcriptional start site (TSS) downstream 500bp and TSS upstream 2000bp) among primate species including human, chimpanzee, gorilla, orangutan, macaque and marmoset. The aligned sequences are the E2F1-specific binding sites located in the promoter region. The promoter sequences of CyclinE1 were not available for most of the nonhuman primate species. For Caspase 7, the promoter sequences of chimpanzee and marmoset were not available. The numbers for positions are the distances from the transcriptional start site. [file 1741-7007-11-62-S4.docx]

**Figure S3.** Alignment of the promoter sequences of p73, p107, p18, p27, p14^ARF^, Caspase7 and hTERT (genes’ transcriptional start site (TSS) downstream 500bp and TSS upstream 2000bp) among primate species including human, chimpanzee, gorilla, orangutan, macaque and marmoset. The aligned sequences are the E2F1-specific binding sites located in the promoter region. The promoter sequences of CyclinE1 were not available for most of the nonhuman primate species. For Caspase 7, the promoter sequences of chimpanzee and marmoset were not available. The numbers for positions are the distances from the transcriptional start site.

**p73**

**TERT**

**
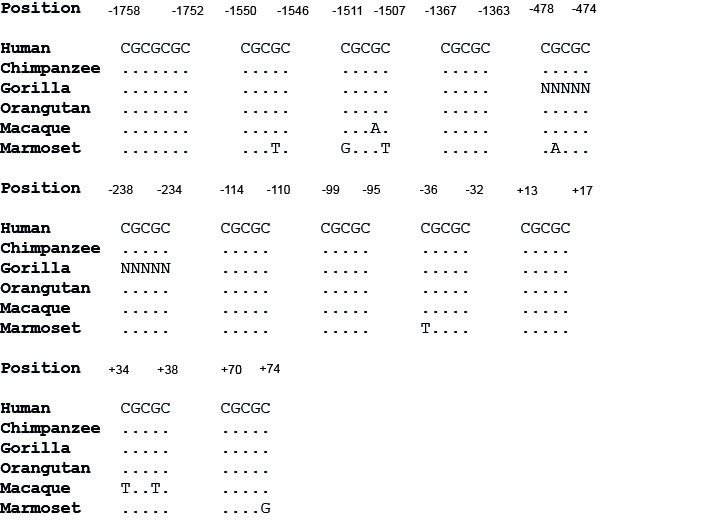

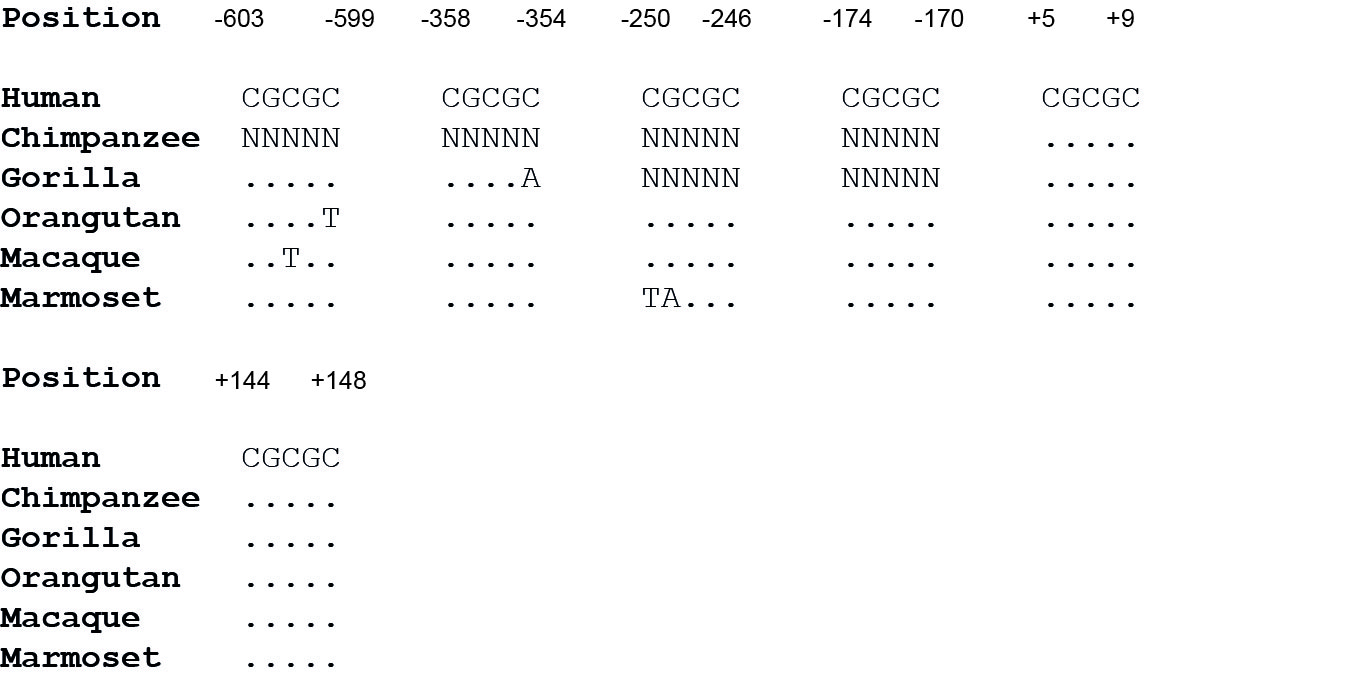
**

**p18 p27 p14^ARF^ Caspase 7 p107**

**
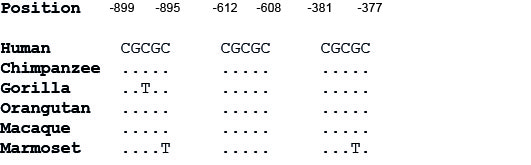

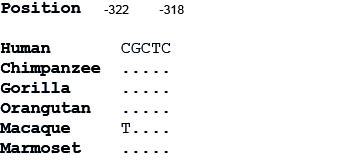

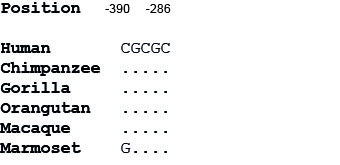

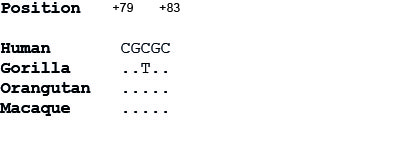

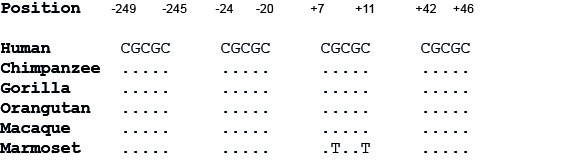
**
