# Supplementary material for: Functional divergence of the brain-size regulating gene MCPH1 during primate evolution and the origin of humans
Source: BMC Biol. 2013 May 22;11:62. doi: 10.1186/1741-7007-11-62 (PMC3674976; doi:10.1186/1741-7007-11-62)
Supplement: Additional file 5: Figure S4 — Alignment of the full length E2F1 protein sequences among different primate species including human, chimpanzee, gorilla and macaque. [file 1741-7007-11-62-S5.docx]

**Figure S4.** Alignment of the full length E2F1 protein sequences among different primate species including human, chimpanzee, gorilla and macaque.

**
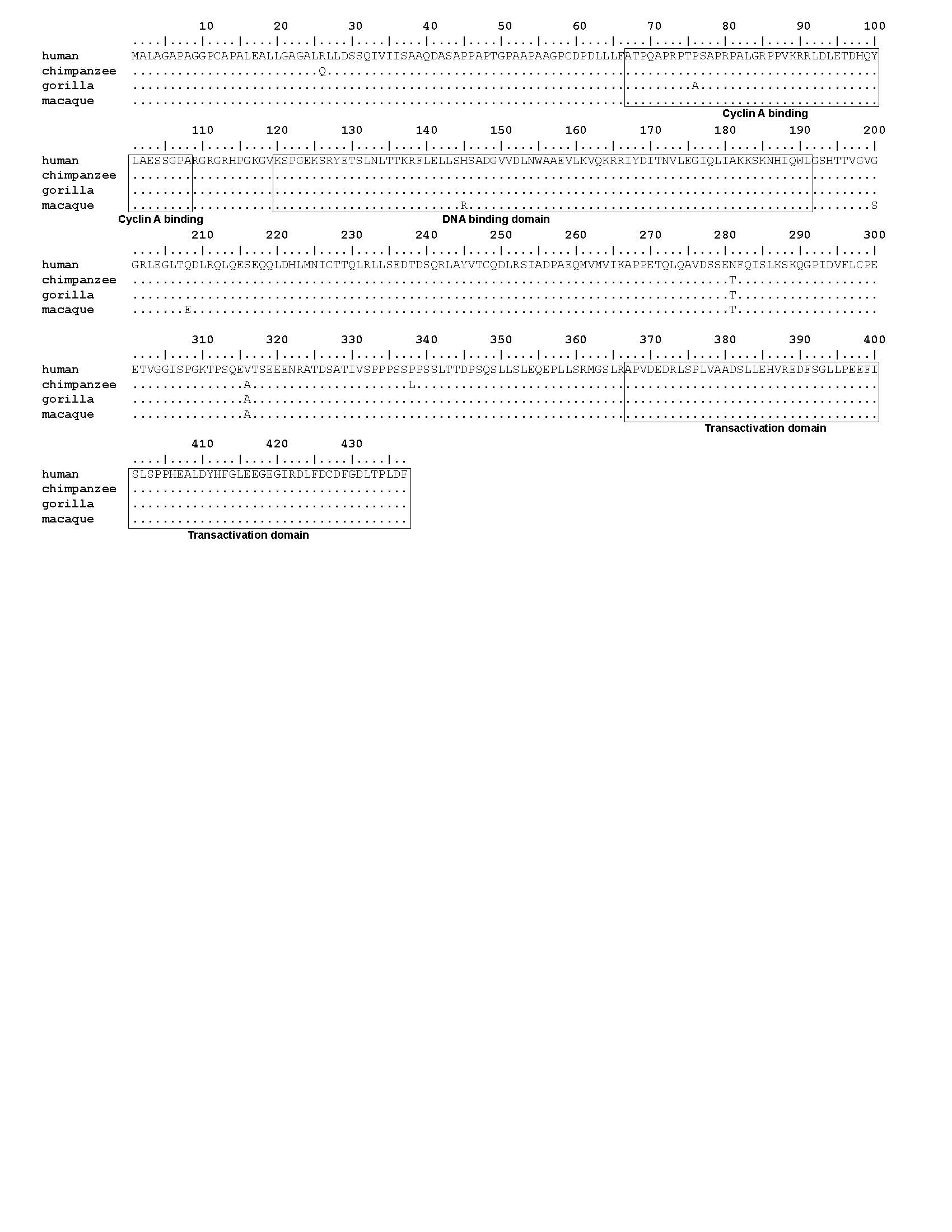
**
