# Supplementary figures and images for: Functional divergence of the brain-size regulating gene MCPH1 during primate evolution and the origin of humans
Source: BMC Biol. 2013 May 22;11:62. doi: 10.1186/1741-7007-11-62 (PMC3674976; doi:10.1186/1741-7007-11-62)

**Figure S5.** The results of the enhancing assay for the E2F1 target genes p18, p27, p107 and Caspase7.


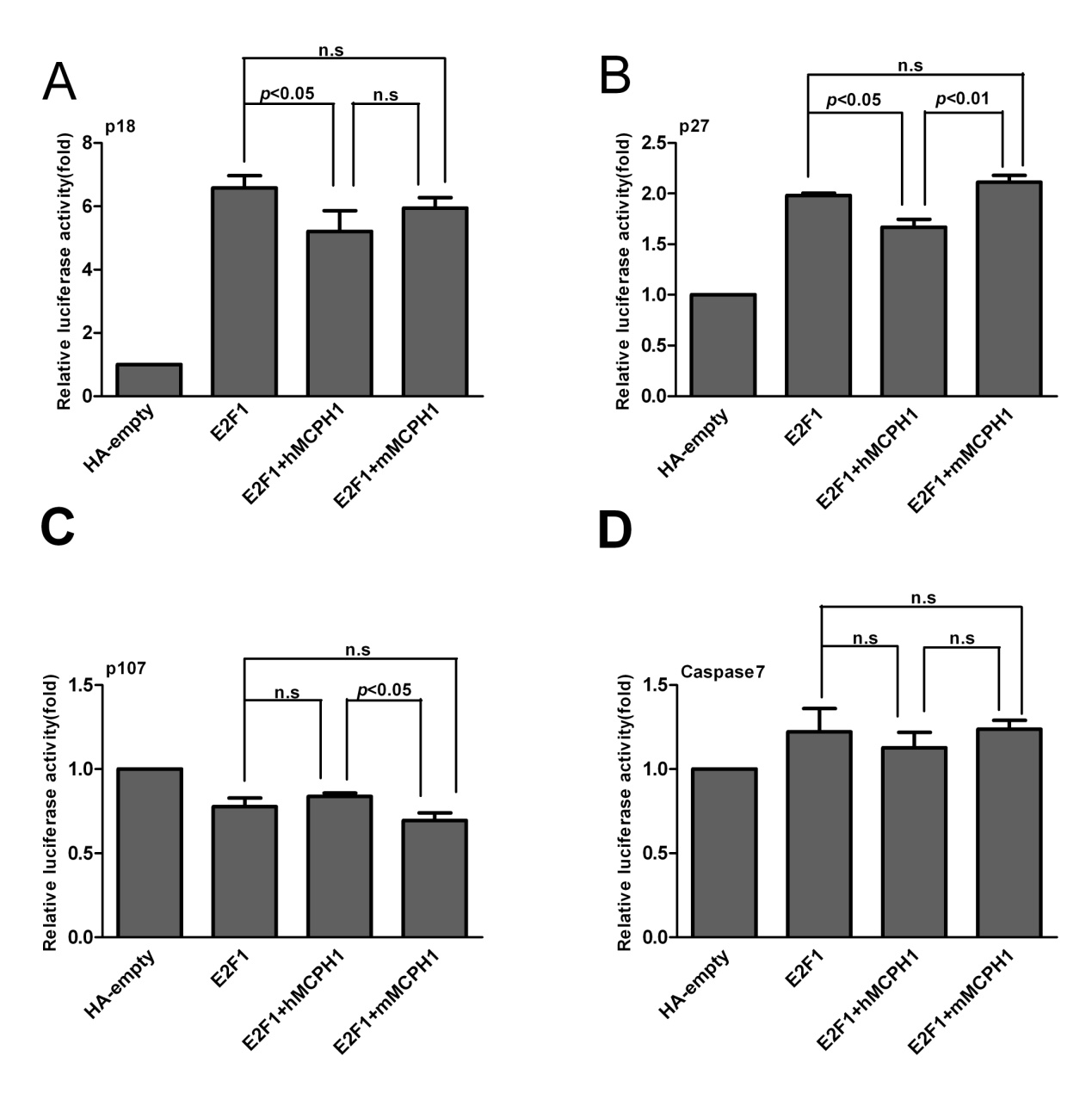

Supplement: Additional file 6: Figure S5 — The results of the enhancing assay for the E2F1 target genes p18, p27, p107 and Caspase7. [file 1741-7007-11-62-S6.docx]
