# Supplementary material for: Functional divergence of the brain-size regulating gene MCPH1 during primate evolution and the origin of humans
Source: BMC Biol. 2013 May 22;11:62. doi: 10.1186/1741-7007-11-62 (PMC3674976; doi:10.1186/1741-7007-11-62)

**Figure S6**. The results of the repressing assay for the target genes including p18, p27, p107, Caspase7 and TERT.


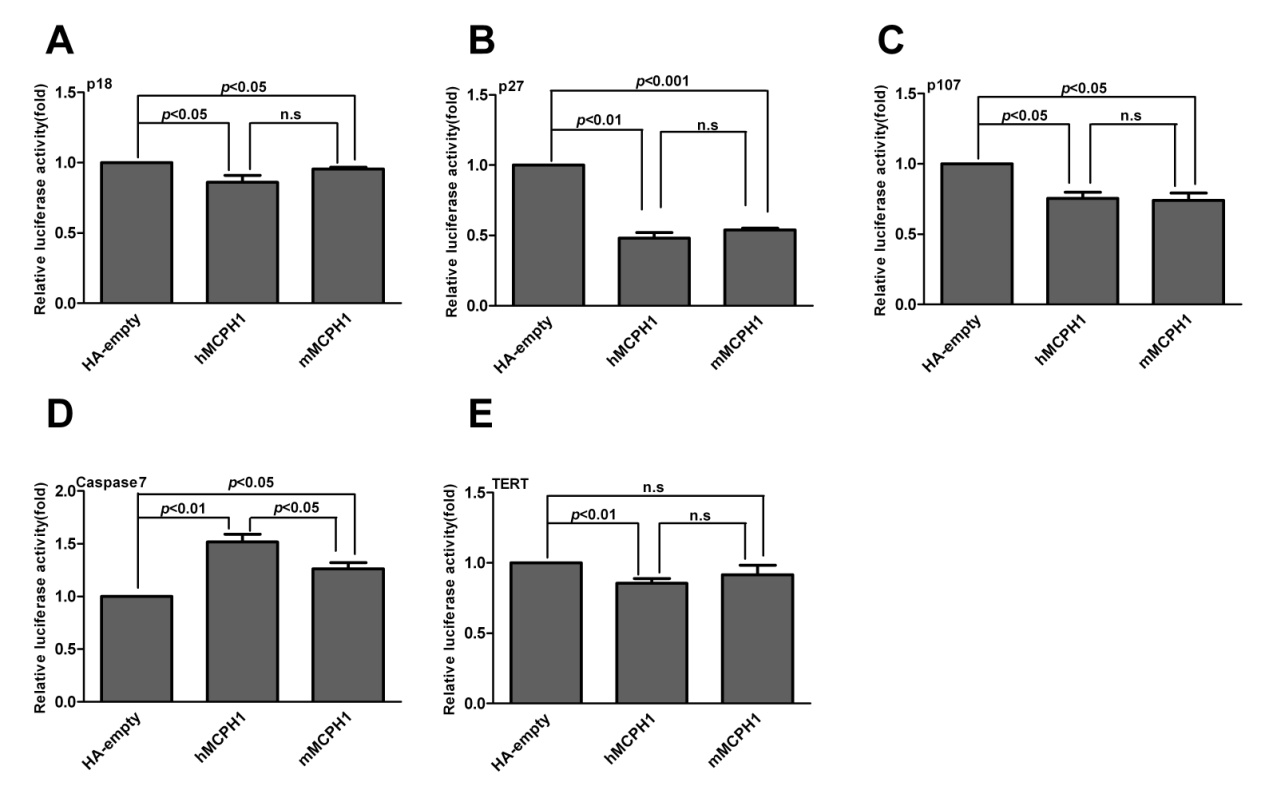

Supplement: Additional file 7: Figure S6 — The results of the repressing assay for the target genes including p18, p27, p107, Caspase7 and TERT. [file 1741-7007-11-62-S7.docx]
