# Supplementary material for: Functional divergence of the brain-size regulating gene MCPH1 during primate evolution and the origin of humans
Source: BMC Biol. 2013 May 22;11:62. doi: 10.1186/1741-7007-11-62 (PMC3674976; doi:10.1186/1741-7007-11-62)
Supplement: Additional file 8: Table S2 — Primers used for the generation of human-specific mutants. [file 1741-7007-11-62-S8.docx]

**Table S2.** Primers used for the generation of human-specific mutants.

| Primer ID | Sequence |
| --- | --- |
| MCPH1 96 _sense  MCPH1 96 _antisense  MCPH1 101 _sense  MCPH1 101 _antisense  MCPH1 310 _sense  MCPH1 310 _antisense  MCPH1 314 _sense  MCPH1 314 _antisense  MCPH1 377 _sense  MCPH1 377 _antisense  MCPH1 425 _sense  MCPH1 425 _antisense  MCPH1 442 _sense  MCPH1 442 _antisense  MCPH1 485 _sense  MCPH1 485 _antisense  MCPH1 835 _sense  MCPH1 835 _antisense | 5GTTCCCTGCAGCTAATTTGAATGAACACTTATCA3  5TGATAAGTGTTCATTCAAATTAGCTGCAGGGAAC3  5TATGAATGAACACTTACCAAGCCTAATTAAAAAAAAACG3  5CGTTTTTTTTTAATTAGGCTTGGTAAGTGTTCATTCATA3  5AATATTGCAGGTAAAATAGTCACCCCTCACC3  5 GGTGAGGGGTGACTATTTTACCTGCAATATT3  5GTAAAGTAGTCACCCCTGACCAAAAGCAGGCTGCA3  5TGCAGCCTGCTTTTGGTCAGGGGTGACTACTTTAC3  5GCAAGAGAAAGAGGAGCATCAGGAGATCTATCATGCC3  5GGCATGATAGATCTCCTGATGCTCCTCTTTCTCTTGC3  5 AATCTTAAGGAAAGGAATTCAGAGAATCTTC3  5 GAAGATTCTCTGAATTCCTTTCCTTAAGATT3  5CAAGCCCTGCTCAGTTTAGCTGCAGAAGTCTT3  5AAGACTTCTGCAGCTAAACTGAGCAGGGCTTG3  5CCATCTCCAGTCCTCGGAAAACTGGAAATGG3  5CCATTTCCAGTTTTCTGAGGACTGGAGATGG3  5CACAAGGTCTGTGCCTCTGAAAACTACCTATTGTC3  5GACAATAGGTAGTTTTCAGAGGCACAGACCTTGTG3 |
