# Supplementary material for: Functional divergence of the brain-size regulating gene MCPH1 during primate evolution and the origin of humans
Source: BMC Biol. 2013 May 22;11:62. doi: 10.1186/1741-7007-11-62 (PMC3674976; doi:10.1186/1741-7007-11-62)
Supplement: Additional file 9: Table S3 — Primers used for the generation of great-ape-specific mutants. [file 1741-7007-11-62-S9.docx]

**Table S3.** Primers used for the generation of great-ape-specific mutants.

| Primer ID | Sequence |
| --- | --- |
| MCPH1 161 _sense  MCPH1 161 _antisense  MCPH1 167 _sense  MCPH1 167 _antisense  MCPH1 510 _sense  MCPH1 510 _antisense  MCPH1 841 _sense  MCPH1 841 _antisense | 5GAATCTAATGGTTCATTAACATATACTCCCACAATTG3  5CAATTGTGGGAGTATATGTTAATGAACCATTAGATTC3  5TATACTCCCACAATTAAAATTAATAGTAGTCACCACAGCGC3  5GCGCTGTGGTGACTACTATTAATTTTAATTGTGGGAGTATA3  5TCTGCCCCTGAAGAAACCCTAAGGTGTTGTAGACAG3  5CTGTCTACAACACCTTAGGGTTTCTTCAGGGGCAGA3  5TACCTATTGCCACAATGAACCGGTCGCGGCC3  5GGCCGCGACCGGTTCATTGTGGCAATAGGTA3 |
